# Recombination and biased segregation of mitochondrial genomes during crossing and meiosis of different *Schizosaccharomyces pombe* strains

**DOI:** 10.17912/micropub.biology.000390

**Published:** 2021-05-17

**Authors:** Stephan Kamrad, María Rodríguez-López, Shoumit Dey, Mimoza Hoti, Henry Wallace, Markus Ralser, Jürg Bähler

**Affiliations:** 1 University College London, Institute of Healthy Ageing and Department of Genetics, Evolution & Environment, London, U.K.; 2 The Francis Crick Institute, Molecular Biology of Metabolism Laboratory, London, U.K.; 3 Charité Universitätsmedizin Berlin, Institute of Biochemistry, Berlin, Germany

## Abstract

During meiosis, tethering of parental mitochondria to opposite cell poles inhibits the mixing of mitochondria with different genomes and ensures uniparental inheritance in the**standard laboratory strain of fission yeast. We here investigate mitochondrial inheritance in crosses between natural isolates using tetrad dissection and next-generation sequencing. We find that colonies grown from single spores can sometimes carry a mix of mitochondrial genotypes, that mitochondrial genomes can recombine during meiosis, that in some cases tetrads do not follow the 2:2 segregation pattern, and that certain crosses may feature a weak bias towards one of the parents. Together, these findings paint a more nuanced picture of mitochondrial inheritance in the wild.

**Figure 1. Mitochondrial inheritance in colonies derived from single spores investigated by next-generation sequencing:**
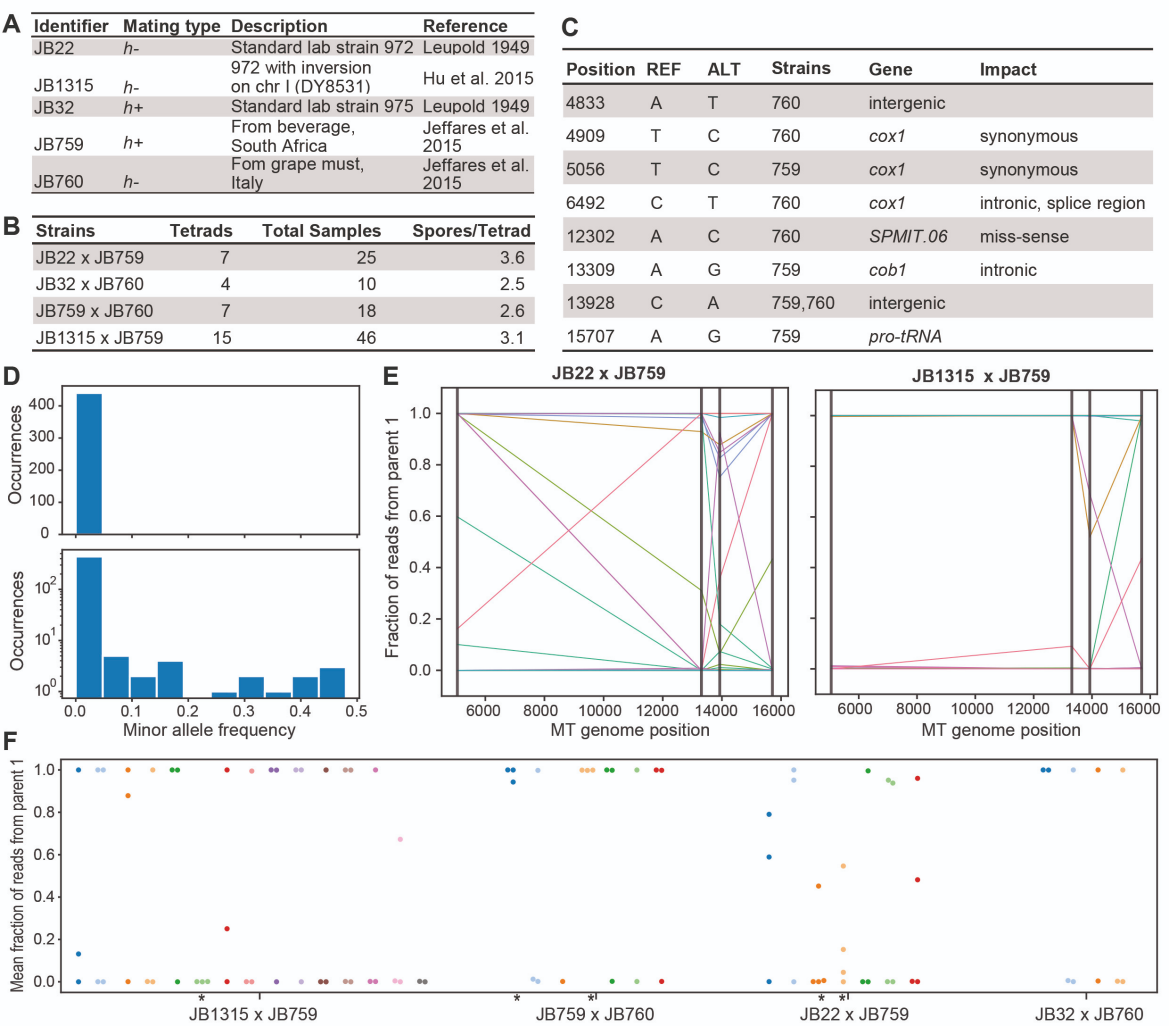
**(A)** Overview of strains used in this study. The wild strains are genotypically and phenotypically distinct from the standard lab strains. **(B)** Overview of crosses made in this study. We crossed strains of opposite mating type and dissected tetrads. Colonies were grown from single spores and genotyped by next-generation sequencing. **(C)** Overview of single-nucleotide polymorphisms across mitochondrial genomes of parental strains shown in (A). These variants allow for the assignment of spore mitochondrial genomes to either parent and the detection of recombination events. The Strains column lists the strains carrying the non-reference allele. **(D)** Fractions of minor observations across all variable sites and samples; *i.e.* a value of 0.5 (x-axis) indicates that alleles from both parents are equally abundant in that locus and sample. These data indicate that colonies containing a mix of both parental alleles can be observed, although they are rare. Both graphs show the same data on different scales. **(E)** Fractions of observations (aligned reads passing quality filters and assigned to one of the alleles) assigned to Parent 1 for four positions across the mitochondrial genome (indicated by grey vertical lines). Each colored line represents a colony grown from a single spore. Some strains showed clear evidence of crossover events. **(F)** Mean fraction of observations assigned to Parent 1 (averaged across all variants) for each colony grown from a single spore. Colors indicate spores belonging to the same tetrad. Tetrads marked with asterisks indicate cases that deviate from the expected 2:2 segregation. Data points falling between 1 and 0 indicate recombination of mitochondrial genomes.

## Description

Mitochondria are typically inherited from one parent only, which is thought to suppress selfish genetic elements. In organisms with differentiated sexes, mitochondria are usually inherited through the mother (Hoekstra 2000). Due to the larger size of the maternal gamete, paternal mitochondria naturally find themselves at a disadvantage and they are further excluded, sequestered and/or degraded by dedicated mechanisms (Bendich 2013). Yeasts have no sexes in the strict sense as their gametes are of equal size (isogamy), and mating types do not determine mitochondrial transmission. Yet, yeasts have been shown to inherit mitochondrial genomes largely uniparentally. *Saccharomyces cerevisiae* mitochondria from both parents are mixed during gamete fusion but interactions with the cytoskeleton and bottleneck mechanisms ensure that only a single mitotype persists after a few rounds of mitotic division (Xu and He 2015). A recent paper has elucidated the mechanism through which uniparental inheritance is achieved in the standard laboratory strain of *Schizosaccharomyces pombe* (Chacko, Mehta, and Ananthanarayanan 2019). Mitochondria are anchored to the cell poles by Mcp5 and stay physically separated during meiosis, ultimately resulting in spores inheriting only those mitochondria anchored on their side of the tetrad. Each parent, therefore, passes on its mitochondria to only two of the four spores.

We wondered whether this mechanism also applies to natural *S. pombe* isolates. To this end, we set up crosses between standard laboratory strains and two strains from our collection of wild strains (Jeffares *et al.* 2015). Tetrads were then dissected, individual spores were grown into colonies and then subjected to whole-genome shotgun sequencing. Unlike the fluorescent labelling approach (Chacko, Mehta, and Ananthanarayanan 2019), this approach does not visualise mitochondria from each parent in real-time, but it is more quantitative and can reveal genetic recombination events and rare alleles.

We set up three crosses between the standard laboratory strains with mating type *h^–^* (JB22) and mating type *h^+^*(JB32) and two wild strains (JB759 isolated from Italy, and JB760 isolated from South Africa) (Fig. 1A). We also set up crosses between JB759 and JB1315. The latter strain is largely isogenic to JB22 except for a large chromosomal inversion on chromosome I to mirror the inversion in JB759 (Hu, Suo, and Du 2015), which should improve spore viability (Avelar et al. 2013). We dissected tetrads and genotyped by sequencing a total of 99 colonies, each originating from a single spore (Fig. 1B). We additionally included the parental strains as controls. While the typical sequencing depth of the nuclear genome was low (~2-6x), the coverage of the mitochondrial genome was much higher (~200-1000x), allowing for highly precise genotyping. Focusing on the mitochondrial genome only, we aligned reads to the reference genome and called variants for each of the samples (Methods, Extended Data 1). We found a total of 8 variants between the parental strains which passed quality filters (Fig. 1C), allowing us to distinguish mitochondrial genomes originating from different parents.

We first looked for evidence of heteroplasmy, i.e. the presence of two mitochondrial genotypes in the same strain. For this, we queried observation counts (number of aligned reads passing quality filters assigned to each allele) for reference and alternative alleles obtained during variant calling (Methods). Surprisingly, we found several strains displaying evidence for heteroplasmy, albeit these were generally rare (Fig. 1D). Overall, 4.3% of variant calls showed alternate observation fractions above 5%. As we sequenced colonies grown from a single spore, rather than individual cells, there could be several explanations for this finding: (i) individual cells maintained mitochondrial genomes from both parents (heteroplasmy), although this seems unlikely based on observations in other organisms; (ii) individual cells were still in the process of ‘sorting out’ their mitochondria and only one genotype will persist after more mitotic divisions; or (iii) different sub-populations in the colony carried different mitochondrial genomes. In any case, our results reveal that two different mitochondrial genomes can end up in the same spore after meiosis. The three interpretations above differ in the time that the two mitochondrial genomes co-exist in the cells generated by this spore through mitotic divisions. Timecourse experiments and re-streaking of single cells could further explore the cause of the observed genetic heterogeneity. Notably, we did not observe a single colony that showed a constant allele frequency across all variant positions except 1 or 0 (Fig. 1E), indicating that mixed mitochondrial genotypes are associated with recombination.

Mitochondrial genomes of *S. cerevisiae* are known to recombine during mating (Fritsch et al. 2014). This would not be expected in *S. pombe* under the model proposed by Chacko et al. (2019) as mitochondria from different parents remain physically separated. For each cross, we plotted observation fractions across the variable loci for each spore. In the two crosses involving strain JB759, we observed several recombination events, i.e. neighboring loci carrying alleles from different parents (Fig. 1E). Eleven colonies from cross JB22 x JB759, four colonies from cross JB1315 x JB759, and one colony from cross JB759 x JB760 showed evidence for recombination. The low number of variants somewhat limits the spatial resolution of recombination events, but if anything this would lead to an underestimation. We conclude that mitochondrial genomes of *S. pombe* can recombine during mating, although it is unclear whether this depends on the parental strains. A different approach would be required to map recombination hotspots as done for budding yeast (Fritsch et al. 2014).

Based on the reported mechanism (Chacko, Mehta, and Ananthanarayanan 2019), the two pairs of spores from a tetrad should each inherit their mitochondrial genome from one parent, reflected in a 2:2 segregation of mitochondrial alleles. Only a minority of tetrads (6 out of 33) in our crosses contained four spores (reflecting the low spore viability observed in crosses involving wild stains; Jeffares et al. 2015). However, among the 33 tetrads dissected in total, we note five cases that appear to violate this pattern as they contain at least three spores that predominantly carry alleles from the same parent (Fig. 1F). In the crosses involving strain JB759, we also note a possible bias towards inheriting mitochondria from this strain. In all three crosses, the majority of colonies carried mitochondrial genomes predominantly inherited from JB759 (64% in the cross with JB22, 59% in the cross with JB1315, and 67% in the cross with JB760).

We have quantified mitochondrial inheritance in crosses of *S. pombe* wild strains by tetrad dissection and next-generation sequencing. We note that 1) colonies grown from a single spore can sometimes include different mitochondrial genotypes; 2) parental mitochondrial genomes can recombine; and 3) that some tetrads deviate from the expected 2:2 segregation during meiosis. While our findings generally agree with a reported mechanism of meiotic segregation of mitochondria (Chacko, Mehta, and Ananthanarayanan 2019), they provide a refined and more complex picture of mitochondrial inheritance in crosses involving natural isolates. This outcome likely reflects several biological and technical factors. We used crosses between genetically and phenotypically distinct wild strains, and our quantitative sequencing approach is better suited to detect recombination events and genotypic heterogeneity. Limitations of our experiment are that we sequenced only one timepoint of small colonies emerging from single spores, which is not conclusive for stable heteroplasmy, and the sampling of colonies, rather than single cells, which cannot establish whether mitochondrial genotypes co-exist within single cells or in cell different populations within the colony. Nevertheless, our data demonstrate that two different mitochondrial genomes can end up in the same spore after the meiotic divisions. Further experiments will be required to confirm and investigate the transmission bias in the crosses involving JB759. We propose that crosses using natural isolates provide an interesting and useful model to study mitochondrial inheritance in fission yeast.

## Methods

Strain crossing and sampling

Strains were grown on YES-Agar plates and mixed in MEA plates, incubated for 48 hours at 25°C. Once asci were visible, tetrads were dissected using a dissection microscope (MSM400, Singer Instruments) onto YES plates. Spores were incubated at 32°C until colonies reached a size of about 1.5 mm in diameter. In order to assess the segregation patterns, we mostly selected tetrads that contained 3 or 4 viable spores. Cells were collected and stored at -20°C until further processing.

DNA from colonies derived from spores was isolated using the MasterPure Yeast DNA Purification Kit (Epicentre, UK). DNA was sheared to a size of 200-500 nt using a Bioruptor sonicator (Diagenode). Libraries were prepared using the NEBNext Ultra library prep kit (New England Biolabs). QC and quantification of libraries were performed using Bioanalyser (Agilent) and Qubit (Thermo Fisher) instruments. Libraries were sequenced with an Illumina MiSeq instrument using the V3 150 cycle kit. Read data has been deposited in the European Nucleotide Archive under accession number PRJEB44368.

Data analysis

Paired-end reads from a total of 126 samples were aligned to the *S. pombe* reference genome (ASM294v2.30) with *bwa mem* (v 0.7.12, Li 2013), using the -R option to assign read groups and sample names and converted to .bam format using *samtools view -b* (v 1.3; Li et al. 2009)*.* Bam-files were then sorted using *samtools sort* and PCR-duplicates were removed with *samtools rmdup*. Read and alignment quality was checked with *qualimap multi-bamqc* (v 2.2.1; Okonechnikov, Conesa, and García-Alcalde 2016). Seven samples were excluded from further analysis due to low read number or low percentage of reads mapped to *S. pombe*. Variants across all bam files were called in a single freebayes run (v 1.1.0-1-gf15e66e; Garrison and Marth 2012), using the following options: –ploidy 1 –standard-filters –min-coverage 3 –min-alternate-count 3. SNPs within 3 bp of InDels were filtered out using the –SnpGap 3 option of *bcftools filter.* Finally, all sites where over half of the population was not called and calls with a phred score below 30 were excluded using *vcftools* (v 0.1.15; Danecek et al. 2011). Variants were annotated with *SnpEff* (v 4.3i; Cingolani et al. 2012)*.*

VCF files were parsed with a python script and converted to a pandas data frame. We excluded 11 samples (from three separate tetrads) from cross JB22 x JB759 from the analysis as these contained nuclear variants from only one parent (indicative of self-mating). Only biallelic SNPs that were segregating in at least one cross were included in the analysis. Heteroplasmy was quantified for biallelic variants by dividing the AO field by the sum of the AO and RO fields in the case of reference allele calls and RO/(AO+RO) in the case of alternative allele calls. Recombination events were defined as samples with a minor allele frequency greater than 10% at at least one segregating site.

Genotype calls and metadata are provided in Extended Data 1 + 2.

Author Contributions

Stephan Kamrad: Data curation, Investigation, Formal analysis, Writing – review and editing, Writing – original draft; María Rodríguez-López: Investigation, Supervision, Writing – original draft; Shoumit Dey: Investigation, Methodology; Mimoza Hoti: Investigation; Henry Wallace: Investigation; Markus Ralser: Funding acquisition, Supervision; Jürg Bähler: Conceptualization, Funding acquisition, Project administration, Supervision, Methodology, Writing – review and editing.
